# Pulmonary metastases of a borderline ovarian tumor with multiple cystic formations: a case report

**DOI:** 10.1186/s40792-023-01616-9

**Published:** 2023-03-08

**Authors:** Rintaro Hamada, Yo Tsukamoto, Makoto Odaka, Mitsuo Yabe, Rintaro Shigemori, Tadashi Akiba, Naoki Toya, Takashi Ohtsuka

**Affiliations:** 1 Department of Surgery, The Jikei University Kashiwa Hospital, 163-1 Kashiwashita Kashiwashi, Chiba, 277-8567 Japan; 2 Department of Surgery, The Jikei University School of Medicine, Nishishinbashi 3-19-18, Minatoku, Tokyo 105-8471 Japan

**Keywords:** Pulmonary metastases, Cyst, Ovarian tumor, Borderline ovarian tumor

## Abstract

**Background:**

Metastatic lung tumors rarely present with cystic formations. This is the first report of multiple cystic formations in pulmonary metastases from mucinous borderline ovarian tumors written in English.

**Case presentation:**

A 41-year-old woman underwent left adnexectomy + partial omentectomy + para-aortic lymphadenectomy for a left ovarian tumor 4 years ago. The pathological finding was mucinous borderline ovarian tumor with a microinvasion. A chest computed tomography performed 3 years after surgery revealed multiple cystic lesions in both lungs. After 1-year follow-up, the cysts increased in size and wall thickness. Subsequently, she was referred to our department with multiple cystic lesions in both lungs. No laboratory findings indicated infectious diseases or autoimmune disorders that could cause cystic lesions in both lungs. Positron emission tomography showed slight accumulation in the cyst wall. Partial resection of the left lower lobe was performed to confirm the pathological diagnosis. The diagnosis was consistent with pulmonary metastases from a previous mucinous borderline ovarian tumor.

**Conclusions:**

This is a rare case of lung metastases from a mucinous borderline ovarian tumor presenting with multiple lesions with cystic formation. Pulmonary cystic formations in patients with a borderline ovarian tumor should be considered as possible pulmonary metastases.

## Background

Although the frequency of cystic formation in primary lung cancer is reported to be 22% [1], it rarely occurs in metastatic lung tumors, accounting for approximately 4% [2]. Thus far, only a few papers have reported cystic formation of lesions in ovarian tumors with pulmonary metastasis [3]. There have been no reports of cystic formations in pulmonary metastases from mucinous borderline ovarian tumors. Herein, we present a rare case of lung metastases from mucinous borderline ovarian tumor (MBOT) presenting with multiple lesions and cystic formation.

## Case presentation

A 41-year-old woman was referred to our department with cystic lesions in both lungs. She underwent left adnexectomy + partial omentectomy + para-aortic lymphadenectomy for a left ovarian tumor at the age of 37 at the Department of Obstetrics and Gynecology of our hospital. She was asymptomatic. The pathological findings were MBOT with a microinvasion. 3 years after the initial surgery, chest computed tomography (CT) revealed multiple cystic lesions in both lungs, with the cysts increasing in size and the cyst walls thickening over time.

**Fig. 1:**
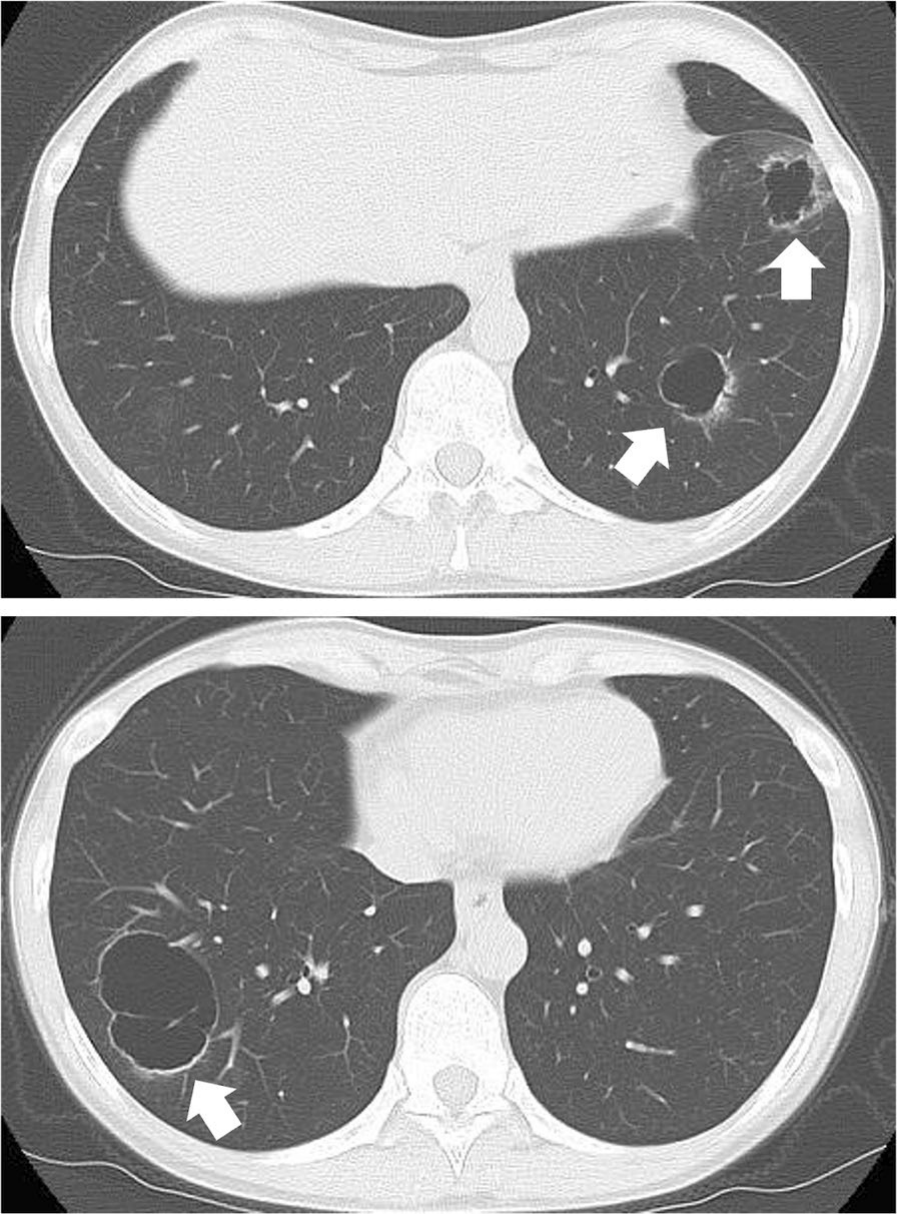
Plain chest computed tomography scans 3 years and 6 months after the initial surgery. Multiple cystic lesions were found in both lungs (white arrow)

**Fig. 2:**
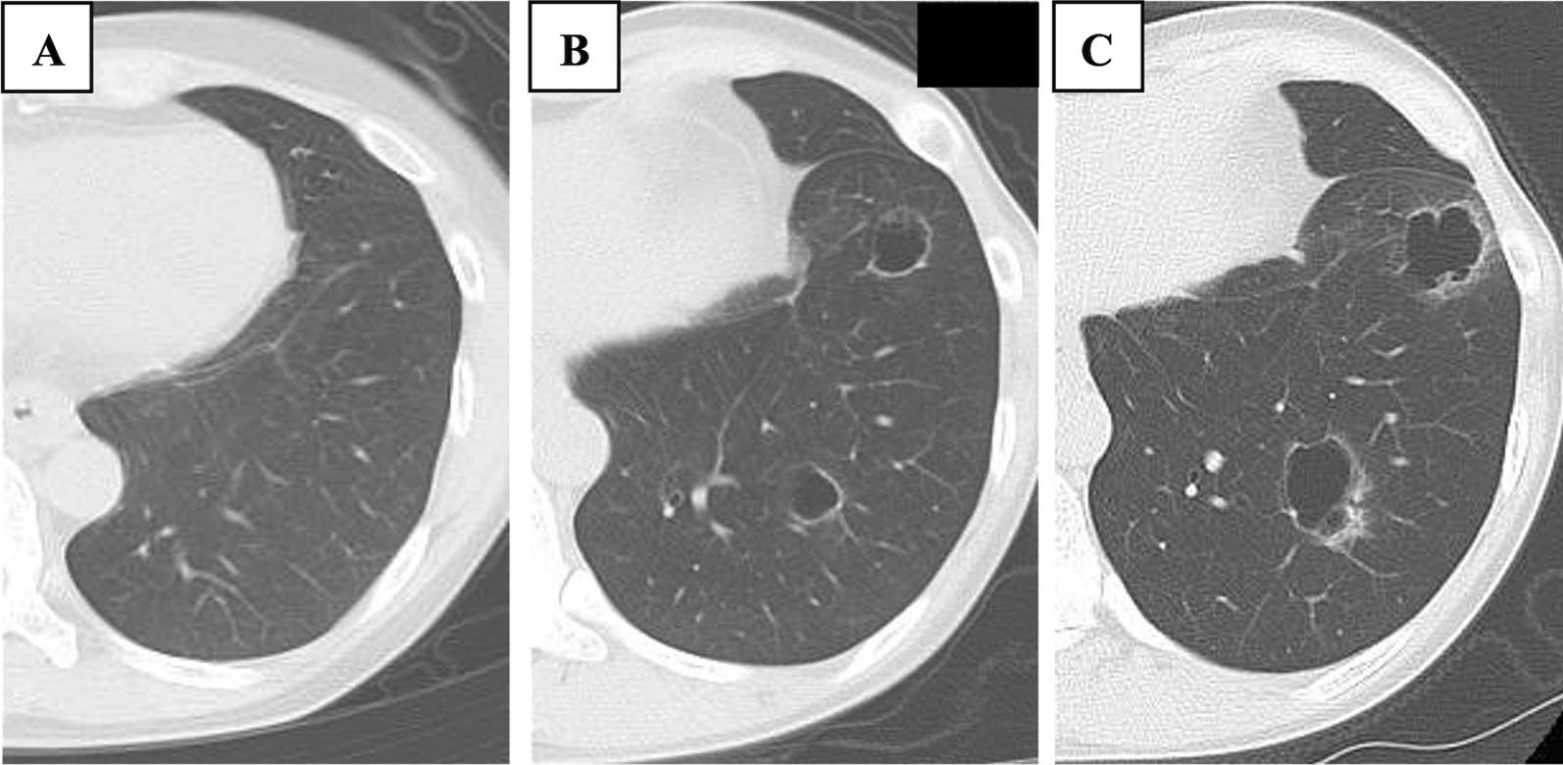
Changes on plain chest computed tomography. The cysts gradually enlarged and their walls were thickened. **A** 2 years after the initial surgery, **B** 3 years after the initial surgery, and **C** 3 years and 6 months after the initial surgery

She had no specific family history, was a social drinker with no smoking history, and took potassium gluconate and heavy magnesium oxide. A plain chest CT revealed multiple cystic lesions in both lungs (Fig. 1). After 1-year follow-up, the cysts were enlarging and their walls thickening (Fig. 2). Blood tests showed no abnormalities in complete blood count, serum chemistries, or coagulation studies. The concentrations of tumor markers, which were carcinoma embryonic antigen, cancer antigen 19-9, cancer antigen 125, pro-gastrin-releasing peptide, cytokeratin fragment, and soluble interleukin-2 receptor, were within normal limits. Autoantibody tests were negative; therefore, connective tissue disorders were ruled out. No laboratory findings indicated tuberculosis or other infectious diseases that could cause cystic lesions. Positron emission tomography-CT showed slight fluorodeoxyglucose accumulation in the cyst wall. Since the diagnosis was not confirmed by imaging, blood tests, or culture tests, we performed a lung biopsy. Considering the risk of pneumothorax and the possibility of intrapleural seeding, we conducted lung biopsy via thoracoscopy in place of a bronchscopic or a percutaneous biopsy.

**Fig. 3:**
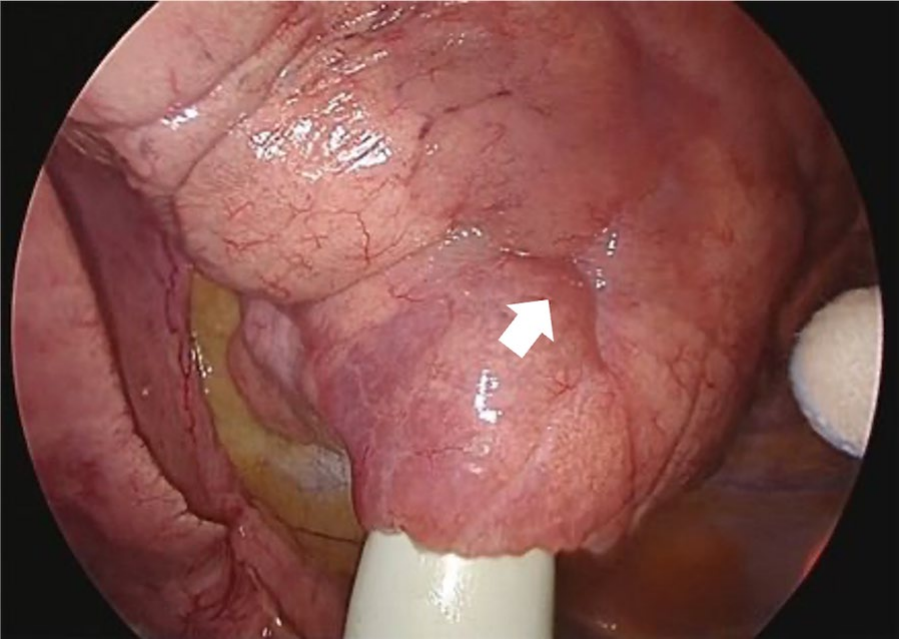
An intraoperative view shows the lesion was palpated as an intrapulmonary cystic nodule with a slightly yellowish tone of the visceral pleura (white arrow)

We performed a complete video-assisted 3-port thoracic surgery. The maximum diameter of the port was 3 cm, and we palpated the tumor by inserting a finger through this port. The tumor was an intrapulmonary cystic nodule with a slightly yellowish tone of the visceral pleura (Fig. 3). We performed a partial resection of the lesion in the left lower lobe, which is where the tumor borders were more easily recognizable.

**Fig. 4:**
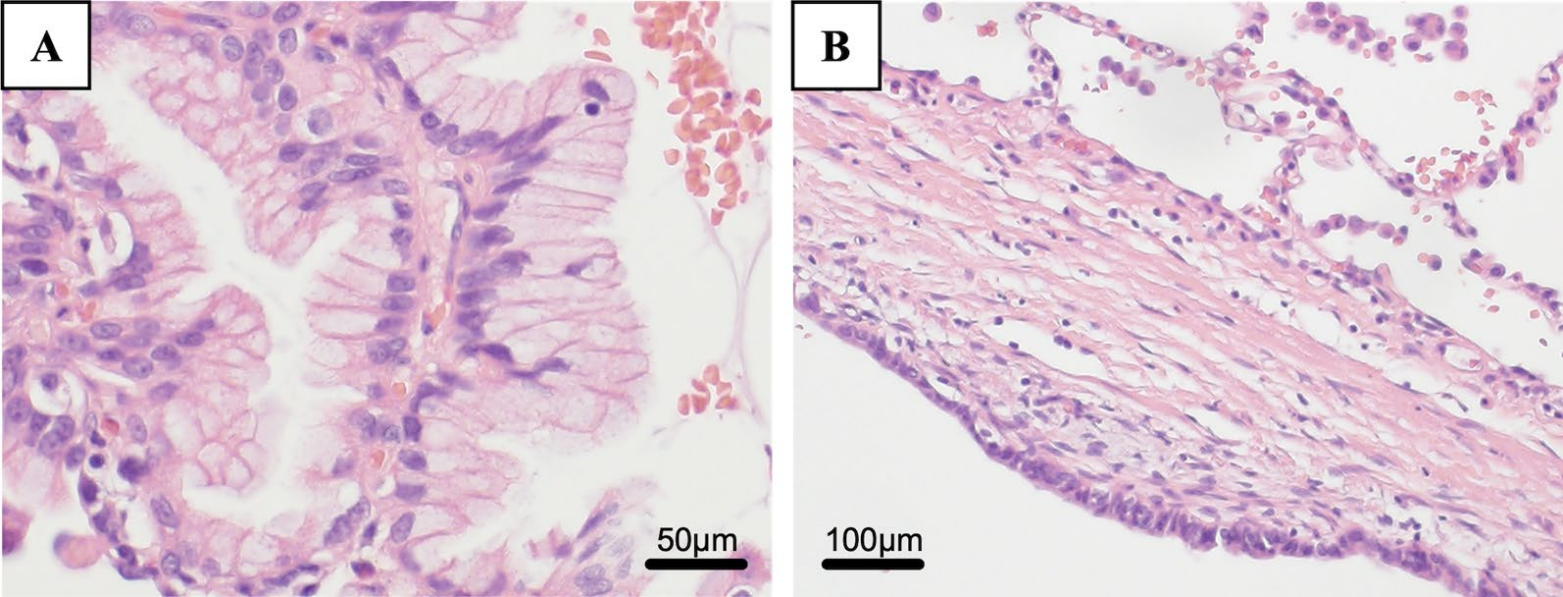
**A** In one area of the tumor, poor atypical glandular epithelium with abundant intracytoplasmic mucus proliferated, forming as papillary and tubular structures (× 200). **B** In the other area, the fibrous stroma was covered with mucus-bearing epithelium forming a septum (× 100)

**Fig. 5:**
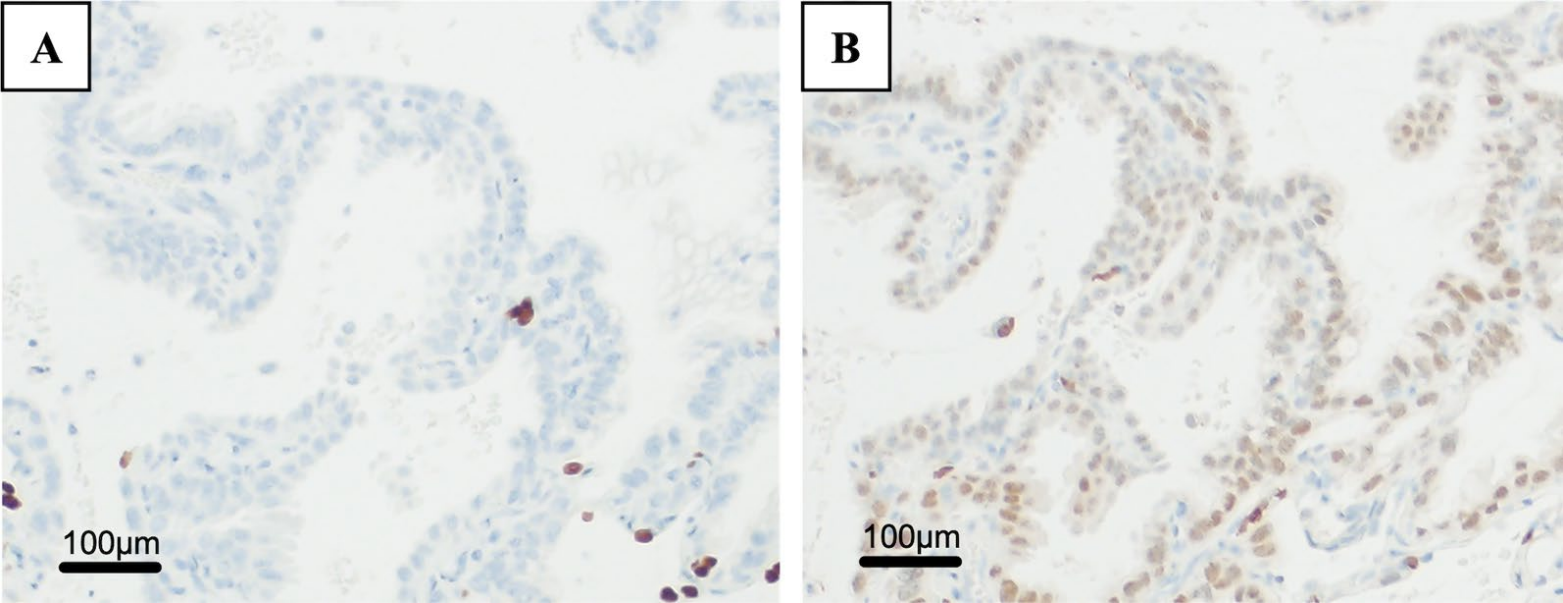
Macroscopic images of the resected lung metastasis by low power field. **A** Negative for thyroid transcription factor-1 staining (× 100). **B** Positive for paired box 8 staining (× 100)

Histopathologically, the cyst was a multilocular lesion with a septum, and there was a hemorrhage within the cyst. As regards the primary focus, the cyst was coated with mucus-filled epithelium, and its cavity was filled with liquid. Although nuclear atypia and cellular stacking were observed, no infiltration was observed. Tumors were found within the cystic structures, and two distinct histological structures were observed within the tumor. One area had poor atypical glandular epithelium with abundant intracytoplasmic mucus that proliferated as forming papillary and tubular structures (Fig. 4A), and the other had fibrous stroma covered with mucus-bearing epithelium forming a septum (Fig. 4B). Various immunostains were negative for thyroid transcription factor-1, which is positive in lung adenocarcinoma (Fig. 5A). On the contrary, paired box 8 staining, which is specific to the female genitalia, was positive (Fig. 5B). Based on these findings, the patient was diagnosed with pulmonary metastases of an MBOT.

We recommended 6 courses of paclitaxel and carboplatin, but the patient refused them, so we followed the patient with CT. On imaging, the cystic lesion tended to enlarge, but no enlarged lymph nodes suggested metastasis nor distant metastasis. 1 year after video-assisted thoracic surgery, the patient was still asymptomatic and visits our outpatient clinic.

## Discussion

We performed a lung biopsy via thoracoscopic surgery to confirm the diagnosis of multiple pulmonary cystic lesions in a patient previously treated for an ovarian tumor.

BOT is a histopathological intermediate between benign cystadenoma and invasive carcinoma and undergoes various morphologic changes. Common subtypes are serous BOT and MBOT. In Asia, MBOT is the most common type of BOT [4, 5]. BOT accounts for approximately 10%–20% of all ovarian neoplasms [6] and has a good prognosis. However, 4%–20% of patients suffer from recurrent or persistent BOT and malignant transformation following initial treatment [7, 8]. Although cystic formation occurs in 22% of primary lung cancer cases [1], it is rarely found in metastatic lung tumors (about 4%) [2]. To the best of our knowledge, no studies have reported cases of multiple lesions with a cystic formation in lung metastases from MBOT.

Pulmonary diseases with a cystic formation are broadly classified into infectious and noninfectious. Infectious diseases include lung abscesses, tuberculosis, nontuberculous mycobacterial infection, pulmonary aspergillosis, and septic embolism. On the contrary, noninfectious diseases include tumors, autoimmune diseases (such as granulomatosis with polyangiitis), and amyloidosis [2].

Regarding the mechanism of multiple metastases from a nonmalignant tumor, haematogenous is considered highly likely rather than aerogenous, which would be represented by a mucinous bronchioloalveolar cell carcinoma [9]. This is mainly because there were multiple cystic lesions in both lungs, which were discovered first. Furthermore, the characteristic intra-alveolar and intratracheal findings with detached floating cells could not be identified microscopically. Therefore, the possibility of aerogenous metastasis was considered unlikely.

According to the National Comprehensive Cancer Network Clinical Practice Guidelines in Oncology, MBOT with invasive implants should be observed or considered for treatment as a grade 1 (low-grade) serous epithelial carcinoma, which would include intravenous injection of taxane and carboplatin for 3–6 cycles as chemotherapy [10]. Since chemotherapy has not been shown to be beneficial in MBOT with invasive implants, further clinical trials are warranted.

The following mechanisms have been proposed to explain cystic formation in metastatic pulmonary lesions [11–13]. First, malignant cells have invaded the wall of a preexisting benign lung cyst. Second, tumor tissues undergo ischemic necrosis, melt, absorbed, or discharged from the induced bronchus. Third, tumor-induced bronchial infiltration causes a check-bulb mechanism, and a tension cavity is formed. Fourth, an abscess forms based on bronchial obstruction and is drained subsequently. Fifth, mucinous tumors naturally form cysts; their contents are expelled, and a cyst remains.

Histological examination of the patient showed no inflammatory cell infiltration and vasculitis was observed in the cysts of both lungs. This suggested the absence of necrosis and no abscess in the tumors, as second and fourth mechanisms. The cystic formation was considered to have occurred by both the third and fifth mechanisms.

In primary lung cancer, patients with stage I non–small cell lung cancer with cystic lesions are more likely to overexpress epidermal growth factor receptors in their tumors and have a poorer prognosis [14]. However, it was not at all clear whether cystic lesions in metastatic lung tumors could be a prognostic factor. Therefore, further accumulation of cases is expected.

## Conclusions

This is the first report of multiple cystic formations as pulmonary metastases from mucinous borderline ovarian tumors written in English. When a patient with a history of malignancy presents with a cystic lesion in the lungs, further evaluation and treatment should be performed if there is a risk for metastatic and primary lung cancer development.

## Data Availability

All data generated or analyzed during this study are included in this published article.

## Abbreviations

BOT: Borderline ovarian tumor
CT: Computed tomography
MBOT: Mucinous borderline ovarian tumor

## References

[1] Gadkowski LB, Stout JE (2008). Cavitary pulmonary disease. Clin Microbiol Rev.

[2] Grant LA, Babar J, Griffin N (2009). Cysts, cavities, and honeycombing in multisystem disorders: differential diagnosis and findings on thin-section CT. Clin Radiol.

[3] Ma JW, Miao Y, Liang CN, Wang N, Jiang B, Wang QY (2020). Malignant transformation of a borderline ovarian tumor with pulmonary and pleural metastases after years of latency: a case report and literature review. Front Med.

[4] Yasmeen S, Hannan A, Sheikh F, Syed AA, Siddiqui N (2017). Borderline tumors of the ovary: a clinicopathological study. Pak J Med Sci.

[5] Seong SJ, Kim DH, Kim MK, Song T (2015). Controversies in borderline ovarian tumors. J Gynecol Oncol.

[6] du Bois A, Trillsch F, Mahner S, Heitz F, Harter P (2016). Management of borderline ovarian tumors. Ann Oncol.

[7] Sozen H, Vatansever D, Topuz S, Iyibozkurt C, Kandemir H, Yalcin I (2019). Clinicopathological analysis of borderline ovarian tumours and risk factors related to recurrence: experience of single institution. J Obstet Gynaecol.

[8] May J, Skorupskaite K, Congiu M, Ghaoui N, Walker GA, Fegan S (2018). Borderline ovarian tumors: fifteen years’ experience at a scottish tertiary cancer center. Int J Gynecol Cancer.

[9] Gaikwad A, Souza CA, Inacio JR, Gupta A, Sekhon HS, Seely JM (2014). Aerogenous metastases: a potential game changer in the diagnosis and management of primary lung adenocarcinoma. Am J Roentgenol.

[10] Ovarian Cancer Including Fallopian Tube Cancer and Primary Peritoneal Cancer (Version 1. 2019). NCCN Clinical Practice Guidelines in Oncology.

[11] Hasegawa S, Inui K, Kamakari K, Kotoura Y, Suzuki K, Fukumoto M (1999). Pulmonary cysts as the sole metastatic manifestation of soft tissue sarcoma: case report and consideration of the pathogenesis. Chest.

[12] Baba K, Hattori T, Koishikawa I, Kamiya T, Noda A, Kobayashi T (1998). Cavitary pulmonary metastases of gallbladder cancer. Respiration.

[13] Nakamura S (2014). CT Findings of pneumonic adenocarcinoma: comparison between invasive mucinous adenocarcinoma and nonmucinous adenocarcinoma. Glob J Med Res..

[14] Onn A, Choe DH, Herbst RS, Correa AM, Munden RF (2005). Tumor cavitation in stage I non-small cell lung cancer: epidermal growth factor receptor expression and prediction of poor outcome. Radiology.

